# Adaptation, Ecology, and Evolution of the Halophilic Stromatolite Archaeon *Halococcus hamelinensis* Inferred through Genome Analyses

**DOI:** 10.1155/2015/241608

**Published:** 2015-01-29

**Authors:** Reema K. Gudhka, Brett A. Neilan, Brendan P. Burns

**Affiliations:** ^1^School of Biotechnology and Biomolecular Sciences, University of New South Wales, Sydney, NSW 2052, Australia; ^2^Australian Centre for Astrobiology, University of New South Wales, Sydney, NSW 2052, Australia

## Abstract

*Halococcus hamelinensis* was the first archaeon isolated from stromatolites. These geomicrobial ecosystems are thought to be some of the earliest known on Earth, yet, despite their evolutionary significance, the role of Archaea in these systems is still not well understood. Detailed here is the genome sequencing and analysis of an archaeon isolated from stromatolites. The genome of *H. hamelinensis* consisted of 3,133,046 base pairs with an average G+C content of 60.08% and contained 3,150 predicted coding sequences or ORFs, 2,196 (68.67%) of which were protein-coding genes with functional assignments and 954 (29.83%) of which were of unknown function. Codon usage of the *H. hamelinensis* genome was consistent with a highly acidic proteome, a major adaptive mechanism towards high salinity. Amino acid transport and metabolism, inorganic ion transport and metabolism, energy production and conversion, ribosomal structure, and unknown function COG genes were overrepresented. The genome of *H. hamelinensis* also revealed characteristics reflecting its survival in its extreme environment, including putative genes/pathways involved in osmoprotection, oxidative stress response, and UV damage repair. Finally, genome analyses indicated the presence of putative transposases as well as positive matches of genes of *H. hamelinensis* against various genomes of Bacteria, Archaea, and viruses, suggesting the potential for horizontal gene transfer.

## 1. Introduction

Stromatolites are defined as organosedimentary structures produced by the trapping and precipitation of carbonates and sediments as a result of microbial growth and metabolic activity [1]. The presence of stromatolites dates back some 3.5–3.8 billion years in Earth's history, a time period that witnessed the appearance of the first forms of life [2]. The microbial communities that constituted these complex structures also played a significant role in global biogeochemical cycles, including early oxygenation of the atmosphere [3]. Owing to their importance, stromatolites have formed the core of a growing area of microbial ecology and evolution-based research. The most extensive modern stromatolites to date reside in the open marine waters in Exuma Sound in the Bahamas [4] and the hypersaline marine environment of Shark Bay on the western coast of Australia [5, 6].

Previous reports employing a combination of microbial isolation, culture-independent nucleic acid based analyses, and lipid profiling have shown that Shark Bay stromatolites support a significant range of metabolically and phylogenetically diverse microorganisms [5–7]. Major functional groups were identified that are involved in cycling of key nutrients, including oxygenic and anoxygenic photosynthesisers, aerobic heterotrophs, and organisms involved in sulphur cycling. For the first time, a diverse range of Archaea was also identified in the stromatolites of Shark Bay [5–7].

Although most studies to date have focused on the function of cyanobacteria and heterotrophic bacteria in stromatolites, the physiological role and significance of Archaea in stromatolite systems still remains unexplored. Halophilic Archaea in particular may be an important component of the Shark Bay stromatolites vital to ecosystem function. Halophilic Archaea are very well adapted to hypersaline environments and thrive in many different areas, including the Dead Sea and solar salterns [8, 9]. Previously, the first archaeon to be isolated from stromatolites, a Gram-negative, nonmotile, strictly aerobic* Halococcus* isolate (*Halococcus hamelinensis*) was also characterised [10]. This archaeon grows optimally at 37°C in medium containing 15% NaCl, as opposed to 20–30% in other characterized* Halococcus* strains. It metabolizes many simple and complex carbohydrates including glucose, sucrose, xylose, maltose, trehalose, mannitol, galactose, and glycerol [10]. It does not hydrolyze starch or produce indole and is negative for sulfide reduction, urease, and gelatin liquefication, in contrast to other* Halococcus* species. Furthermore, it displays sensitivity to rifampicin, novobiocin, and bacitracin and resistance to kanamycin, tetracycline, streptomycin, neomycin, and penicillin.* H*.* hamelinensis* was also oxidase-negative, whereas all recognized* Halococcus* species are oxidase-positive, and most aerobic halophilic Archaea are also oxidase-positive [11]. Whole-cell protein profiles, enzyme composition, and carbon source utilization of this isolate were distinct from other characterized* Halococcus* species, rendering it a novel species of* Halococcus* [10]. Prior to the isolation of this organism, no archaeal species had been discovered at Shark Bay. As no archaeal isolates were also identified in the surrounding water, despite samples being collected several years apart [5, 7], it may be that this organism has a significant role to play in stromatolite ecosystem function.

Recent studies have shown that* H. hamelinensis* displays a number of other novel characteristics, including lacking potassium accumulation as a primary osmoprotective strategy, in contrast to most known haloarchaea [12]. Instead, accumulation of glycine betaine as well as other solutes such as glutamate and trehalose was observed to be the primary mechanism of adaptation to salt stress in this organism [12]. In addition, a recent study examined the effects of UV stress/damage in* H. hamelinensis* [13], revealing that this archaeon is able to survive high germicidal UVC radiation dosages, as well as possessing several bacterial-like nucleotide excision repair genes [13]. Changes in the expression levels of these genes (uvrA, uvrB, and uvrC) were investigated by qRT-PCR, and all were upregulated during both light and dark repairs [13].

While these recent studies have provided useful insights into the physiological characteristics of this novel archaeon, what is lacking is a comprehensive whole genomic profile and related computational analyses of this organism, which would significantly enhance our understanding of its evolutionary and adaptive traits. Here we describe the sequence (by massively parallel 454 pyrosequencing technology), annotation, and analysis of the genome of* H. hamelinensis*, which provides insights into the ecology, evolution, and adaptation of this novel microorganism.

## 2. Materials and Methods

### 2.1. Genome Sequencing and Assembly

*Halococcus hamelinensis* was grown as previously described until logarithmic phase [10], and DNA was extracted using a xanthogenate protocol previously optimised for this Archaea [14]. The genome of* H. hamelinensis* was sequenced at The Clive and Vera Ramaciotti Centre using 454 (Roche) sequencing technology. Sample quality and quantity were assessed prior to library preparation, as described in the GS FLX Titanium General Library Preparation Method Manual (Roche). Briefly, 8 *μ*g of gDNA was fragmented by nebulization, fragmented DNA run on a 0.8% agarose gel and fragments between ~500 and 800 bp excised, and purified using a QiaQuick Gel Extraction Kit (Qiagen). DNA sample quality assessment, fragment end repair and adaptor ligation, small fragment removal, library immobilization, and single-stranded DNA library isolation were performed as per GS FLX Titanium Sequencing Method Manual (Roche). The quality of the library was assessed on a Bioanalyzer RNA Pico 6000 Chip (Agilent Technologies) and quantified using the Quant-It RiboGreen assay (Invitrogen). Finally emulsion PCR and sequencing were performed as described in the GS FLX Titanium Sequencing Method Manual. A total of 272,854 reads, counting up to 118,638,438 bases (32-fold coverage of the genome), were assembled using 454 Newbler GS* de novo* Assembler software, which generated 453 contigs ranging from 500 to 105,929 bp, with bases having quality scores of 40 and above.

### 2.2. Automated Sequence Annotation

Sequencing output was uploaded onto two different auto annotation servers, the Rapid Annotation using Subsystem Technology (RAST) [15] and Integrated Microbial Genomes/Expert Review (IMG/ER) [16]. RAST uses a strategy based on comparison with manually curated subsystems and subsystem-based protein families, guaranteeing a high degree of consistency and accuracy. Putative coding sequences were identified by Glimmer software [17], and peptides shorter than 30 amino acids were eliminated. tRNA genes and rRNA genes were predicted by tRNAScan-SE software and RNAmmer, respectively [18, 19]. Functional annotation was performed by searching the NCBI nonredundant protein database and the Kyoto Encyclopedia of Genes and Genomes (KEGG) protein database. Data from the genome were matched against data from external sources, including RefSeq, EBI Genome Reviews, GenBank/EMBL, JGI microbial genome data, LIGAND, GO, COG, Enzyme, InterPro, KEGG, KO, Pfam, TIGRFam, and GOLD. Pathways for modes of nutrition and transport system were acquired through the KEGG function analysis in IMG. COG analysis was carried out using COG functions and abundance profile analysis within the IMG/ER.

### 2.3. Bioinformatic Analyses

Phylogenetic analyses were performed to elucidate evolutionary relationships. The 16 S rRNA gene sequences were derived from various euryarchaeal genomes from the NCBI database. The sequences were then aligned using ClustalX [20]. A BLOSUM series protein weight matrix was used with a Gap Opening of 10 and a Gap Extension of 0.2. Using the ClustalX multiple sequence alignment, neighbor joining phylograms were constructed, bootstrapped, and displayed using the MEGA version 4 program [21]. Programs from the EMBOSS package, COMPSEQ (calculates the composition of unique words in a sequence) and CHIPS (calculates Nc codon usage statistics), were run to determine the number and frequency of occurrence of codon triplets of the predicted proteins. Over- or underrepresented codons were corrected based on the GC content (66.08%) of the* H. hamelinensis*. Predictions of amino acid usage were based on the codon frequency output for each amino acid. Radial graphs were created using IMG/ER Phylogenetic Distribution of genes software. The program Alien Hunter was used to detect potential horizontal transfer regions within the* H. hamelinensis* genome. The Alien hunter output was read through the Artemis program and the data output was viewed using the DNA PLOTTER program.

## 3. Results

### 3.1. *H. hamelinensis* Genome Overview

Whole genome sequencing by 454 pyrosequencing produced a greater than 32x coverage of the* H. hamelinensis* genome. The genome sequence of* H. hamelinensis* is accessible at GenBank under accession number PRJNA80845. An overview of the genome features is given in Table 1. The genome of* H. hamelinensis* consisted of 3,133,046 base pairs (bp) with an average G+C content of 60.08%. The genome consisted of 223 DNA scaffolds and 48 RNA genes, 45 of which were tRNA genes and the remaining three were rRNA genes comprising of a single rRNA operon (16 S-23 S-5 S). Furthermore, the genome contained 3,150 predicted coding sequences, 2,196 (68.67%) of which were protein-coding genes with functional assignments and 954 (29.83%) of which were of unknown function. Based on 16 S rDNA phylogenetic analyses,* H. jeotgali* was identified as the closest sequenced genome to* H. hamelinensis* with a BLAST identity score of 91%. Other related organisms were* H. turkmenica*, isolated in sulfate saline soil in Turkmenistan [22],* N. Magadii*, isolates from a Kenyan soda lake [23], and* H. xanaduensis*, isolated from a saline Lake Shangmatala in China [24].

**Figure 1:**
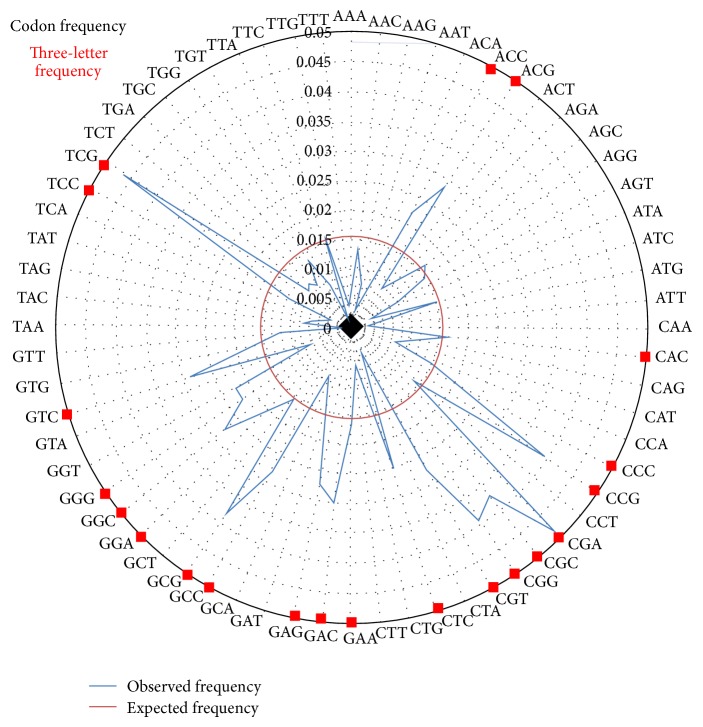
Radial graph displaying the three-letter codon frequency prediction of* H. hamelinensis*. Expected codon frequencies are indicated in the inner circle. Squares in the outer circle are indications are overrepresented codons.

The results for codon frequency indicated a bias towards high GC content in the third position (Figure 1). From the three-letter codon prediction codons, the following amino acids were observed to have high frequency: alanine (GCC, GCG, and GCA), arginine (CGA, CGC, CGG, and CGT), aspartic acid (GAC), glutamic acid (GAA, GAG), glycine (GGC, GGG, and GGT), histidine (CAC), leucine (CTC), proline (CCC, CCG), serine (TCC, TCG), threonine (ACC, ACG), and valine (GTC). Frank Wright's NC statistic for the effective number of codons used was also calculated, where the Nc output can take values from 20, in the case of extreme bias where one codon is exclusively used for each aa, to 61 when the use of alternative synonymous codons is equally likely. The Nc output value for the* H. hamelinensis* genome was calculated as 47.8, indicating very little bias between individual codon usage. Organisms high in GC% tend to have codons ending in C or G and this is also reflected in the* H. hamelinensis* genome.

### 3.2. Carbon Assimilation

Preliminary studies carried out on* H. hamelinensis* whole cells indicated that the organism utilizes glucose, sucrose, xylose, maltose, trehalose, and glycerol as both single and complex carbon sources, in addition to mannitol, galactose and ethanol as single carbon sources [10]. Detailed genome analyses conducted in the present study identified glucose catabolism to proceed via the Embden-Meyerhof pathway. Genes encoding enzymes from the Entner-Doudoroff pathway were not present in the* H. hamelinensis* genome.

### 3.3. Nitrogen Assimilation

Archaea can use ammonia as well as a large variety of nitrogen compounds as sole sources of nitrogen. To assimilate ammonia, only one enzyme is required, glutamine synthetase that was identified here in the* H. hamelinensis* genome (gene ID: 2502298936). Once converted to L-glutamine, the enzyme glutamate synthase (gene ID: 2502300824, 2502301225, and 2502301417) converts it to glutamate. In cases of low ammonia* H. hamelinensis* can putatively convert nitrate to ammonia via nitrite using the enzymes nitrate reductase (catalytic subunit) (gene ID: 2502301552) and ferredoxin nitrite reductase (gene ID: 2502300324). The organism also has the genetic potential to produce glycine and cyclic amidines, using the enzymes aminomethyltransferase/glycine cleavage system T protein (gene ID: 2502301667) and NAD^+^ synthetase (Gene ID: 2502298992, 2502299198, 2502299746). In addition to these enzymes,* H. hamelinensis* also possesses genes encoding three ABC transport proteins involving glutamine, namely, glutamine ABC transporter, periplasmic glutamine-binding protein (gene ID 2502298730), ABC-type glutamine/glutamate/polar amino acids transport system, permease protein (gene ID: 2502298731), and ABC-type glutamine/glutamate polar amino acids transport system, ATP-binding protein (gene ID: 2502298732).

### 3.4. Energy Acquisition

Analyses here confirmed the presence of genes encoding: 3Na^+^/H^+^ antiporters, Na^+^/H^+^ antiporter NhaC, Na^+^/H^+^ antiporter MnhB subunit-related protein, multisubunit Na^+^/H^+^ antiporter, MnhF subunit, and two Na^+^ symporters, namely, Na^+^/solute symporter and pantothenate: Na^+^ symporter, suggesting a functioning pathway between H^+^ and osmotic metabolism. Genome analysis also confirmed the presence of an ATP synthase gene cluster (V-type ATP synthase subunit A, B, C, E, F, H, I, and a ATP synthase subunit C) and putative regulatory, ligand-binding protein related to C-terminal domains of K^+^ channels, indicating complete pathways from H^+^ to K^+^ gradients and ATP production, respectively. Respiratory chain enzymes, in halophilic organisms, require high salt concentration to be active [25]. Genome analyses indicated that* H. hamelinensis* contained a putative electron-transport flavoprotein (alpha and beta subunit) and two cytochrome B coding genes.

The halophilic Archaea possess light driven ion pumps [26], bacteriorhodopsin, and halorhodopsin that pump protons and chloride ions, respectively, across the membrane. The proton pump serves to create a proton potential that can be used to drive ATP synthesis [25]. Although this has been thought to be exclusively an archaeal protein, recently bacteriorhodopsin-like proteins, such as xanthorhodopsin and proteorhodopsin, have been discovered in bacteria [27, 28]. Interestingly, despite exhaustive genome analyses, no homologous genes encoding bacteriorhodopsin, halorhodopsin, or bacterioruberin were identified in the* H. hamelinensis* genome in the present study.

### 3.5. Osmoadaptation

*H. hamelinensis* is known to accumulate compatible solutes such as glycine betaine, trehalose, glutamate, and proline [12], which display a general stabilizing effect by preventing the unfolding and denaturation of proteins caused by heating, freezing, and drying. Genome analyses here revealed several putative genes involved in glycine betaine accumulation processes. The presence of three genes (designated betI, betII, and betIII) encoding for three putative glycine betaine transport proteins supports the previous findings showing glycine betaine accumulation as an osmoprotective strategy in this archeon [12]. Two ABC-type glycine betaine permease components and glycine betaine ABC transporter ATP-binding protein identified here (Table 2) also indicate the presence of a glycine betaine ABC-transport system used to accumulate glycine betaine. In addition, there are two further genes encoding a putative glycine betaine transporter (OpuD) present in the genome. Phylogenetic analysis carried out on the three Bet proteins from* H. hamelinensis* that showed high similarity to putative glycine betaine secondary transporters of other halophilic Archaea as well as those of* Desulfomicrobium baculatum*, ButA of the halophilic bacterium,* Tetragenococcus halophilus*, and OpuD of the marine gamma-proteobacterium,* Teredinibacter turnerae* [29–31]. Twelve transmembrane domains were present, a signature of the BCCT (betaine/carnitine/choline transporter) family. Trehalose is another compatible solute putatively synthesized in* H. hamelinensis* for osmoadaptation [12]. A putative trehalose biosynthesis pathway was found to be complete in the* H. hamelinensis* genome here, where UPD-glucose is metabolised to trehalose-6-phosphate and then trehalose via trehalose 6-phosphate.

Archaea are also known to maintain a constant K^+^ concentration intracellularly for essential functions such as maintenance of cell turgor and homeostasis, adaptation of cells to osmotic stress, and activation of cell cytoplasmic enzymes. There are various mechanisms to accumulate K^+^ in the cell and the three best characterized systems are the Trk, Kdp, and Kup K^+^ transport systems [32]. Previous analyses screening for putative K^+^ uptake genes by degenerative PCR failed to detect two putative potassium transport proteins, namely, TrkH and KdpB, in* H. hamelensis* [12]. While no homologs to the kdpB system were identified in the present study, other genes coding for K^+^ uptake proteins were identified which included two putative K^+^ uptake proteins: TrkH and TrkA (Table 2). These form a part of the Trk system, which is a low affinity, rapid transport system mainly used at neutral or alkaline pH. The presence of homologs to TrkH in the present study is in contrast to PCR results reported recently [12] and illustrates the advantage of whole genome sequencing to complement targeted PCR approaches. Two Kef-type K^+^ transport proteins and multiple sodium-potassium/hydrogen antiporter subunits were also identified here, which comprise the K^+^ efflux system [33]. Interestingly, genes encoding for the synthesis of archaeatidylglyercolmethyl phosphate, a typical lipid identified previously in* H. hamelinensis* [12] and known to contribute to osmotolerance, were not found here.

### 3.6. Strategies of DNA Repair and Heavy Metal Resistance

A recent study revealed the presence of a UvrABC DNA repair system in* H. hamelinensis*, possessing three bacterial-like nucleotide repair genes, namely, excinuclease ABC subunit A, B, and C [13]. Three other putative DNA repair systems were identified in the present study. These were a bacterial MutL-MutS system, consisting of mismatch repair proteins, DNA repair base excision system consisting endonucleases and ligases, and the 2-phosphoglycolate salvage system, consisting of phosphoglycolate phosphatase. In addition there were also several DNA repair and recombination proteins detected within the* H. hamelinensis* genome.

Various Archaea are also known to possess mechanisms for heavy metal resistance. Analyses were conducted here to verify the presence of any toxic/heavy metal resistance coding genes in* H. hamelinensis*. Several putative genes were identified here and are shown in Table 3. Two copper-translocating ATPases were detected, and there was also the presence of genes encoding arsenate reductase enzymes and copper-zinc-cadmium resistance proteins within the* H. hamelinensis* genome. There were four permease enzymes belonging to the drug/metabolite transporter super family, which are presumed to transport toxic metabolites out of the cell.

### 3.7. Cluster of Orthologous Group (COG) Categories

The* H. hamelinensis* genome has a total of 2,296 genes within COG categories. A search was carried out to determine the overrepresented COG categories within* H. hamelinensis*, when compared to other sequenced archaeal genomes. There were 8 overrepresented categories the general prediction category (16.90%), followed by the amino acid transport and metabolism (9.80%), unknown function (9.32%), inorganic ion transport and metabolism (7.62%), energy production and conversion (7.14%) and translation, and ribosomal structure and biogenesis (6.93%). Following these analyses, a search for both homolog and nonhomolog genes between* H. hamelinensis* and Archaea was conducted, and then for the Euryarchaeota. Results indicate a higher number of homolog genes between both Archaea and Euryarchaeota and fewer nonhomolog genes. Genome analysis reveals 165 genes from* H. hamelinensis* that have homologs in the 86 genomes of other Euryarchaeota, whereas there are only 95 genes from* H. hamelinensis* in total that have homologs after comparison with all other 120 Archaea genomes.

### 3.8. Potential Horizontal Gene Transfer

Nine putative transposase coding genes were detected in the* H. hamelinensis* genome. These were distributed amongst six different contigs. Two transposase DDE domains were found close to each other on contig 00141. Neighbouring these transposase genes were three genes, two ABC transport protein coding genes and one putative cobalamin protein coding gene, from the inorganic ion transport and metabolism COG category. Two putative transposase coding genes are found in close proximity to a glycine betaine transporter and a glycine cleavage T-protein. Other genes neighbouring the transposases are genes belonging to the general function prediction COG category as well as the inorganic ion transport and metabolism COG category. A third putative transposase DDE domain was found isolated on contig 00142 with a hypothetical protein neighbouring it. A phylogenetic distribution of genes was assessed by carrying out individual BLAST searches between each protein coding gene of* H. hamelinensis* and genes across all other phyla. This was conducted to analyze potential horizontally transferred genes. It was found that* H. hamelinensis* has 2,639 positive matches within the archaeal lineage, out of which 96.89% fall within the Euryarchaeota phylum and 117 hits within the bacterial lineage. Under the bacterial lineage, the highest numbers of genes with positive hits were against the following: the Actinobacteria phylum (22.%), Bacilli (15%), Gammaproteobacteria (11%), Clostridia (9%), Cyanobacteria (8%), and Bacteroidetes (7%).

**Figure 2:**
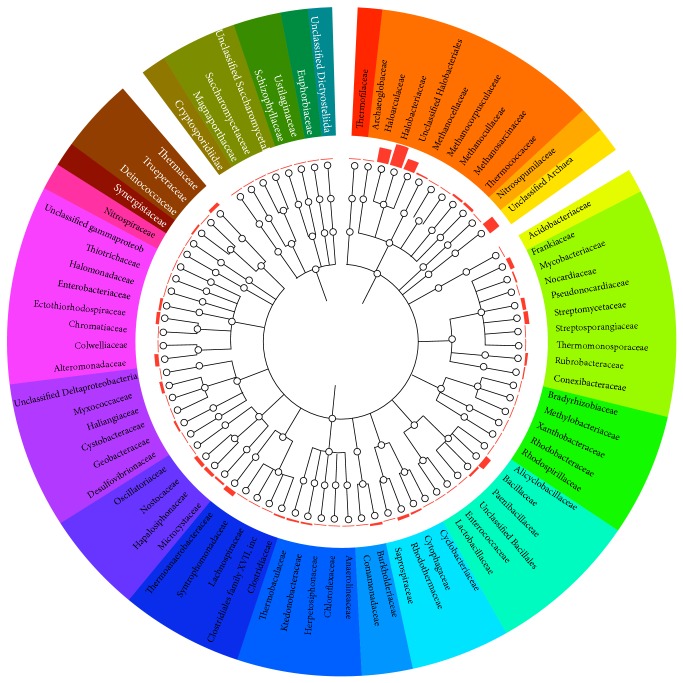
Radial phylogenetic tree comparison between* H. hamelinensis* and the bacterial and archaeal lineage within IMG/ER database. Genomes of Archaea, Bacteria, and Eukaryota are displayed clockwise on the circular plot. The bar charts in between the circular plot and phylogenetic dendrograms are percentage representations of positive hits of genes between* H. hamelinensis* and respective genomes.

A radial phylogenetic tree comparison between* H. hamelinensis* and other bacteria and Archaea (Figure 2) also indicate potential evolutionary relationships and/or potential HGT that may have occurred. To further examine the importance of potential horizontal gene transfer to the* H. hamelinensis* genome, a gene-independent analysis of potential HGT using interpolated variable motif scores calculated from the program Alien Hunter was performed. Several putative regions were considered likely to have been acquired by HGT (data not shown).

### 3.9. Screening for Restriction/Modification and CRISPR Systems in* H. hamelinensis*

Microbial defence systems against mobile elements/foreign DNA include restriction modification (RM) systems [34] and clustered, regularly interspaced, short palindromic repeat (CRISPR) systems [35]. CRISPR systems have been recently identified as an adaptive microbial immune system that provides acquired immunity against viruses and plasmids. This system represents a family of DNA repeats found in approximately 40% of bacterial genomes and most archaeal (~90%) genomes thus far screened [35]. Interestingly, after analysis of the* H. hamelinensis* genome no putative CRISPR/Cas elements could be identified. RM systems were also examined by screening the* H. hamelinensis* annotated genome for genes involved in RM systems; no homologs to known host restriction modification systems were identified and only one homolog to a methylase putatively involved in methylating (and thus protecting) host DNA from host restriction enzymes (CTAG modification methylase) was identified.

## 4. Discussion

The present study for the first time presents detailed bioinformatic analyses of the* Halococcus hamelinensis* genome. This microorganism was originally isolated from modern stromatolites in the hypersaline reaches of Shark Bay, Australia, and is known to exhibit several novel characteristics [10, 12]. However, few genomes of stromatolite organisms have been fully sequenced and are available for comparative genomics. Furthermore, to date little is known in regards to the adaptive mechanisms employed by Archaea that reside in such ecosystems, and the exact role of Archaea in stromatolites remains clouded. To better understand the basis of survival and potentially any role* H. hamelinensis* may play in its unique environment, the genome was sequenced, annotated, and detailed bioinformatics analyses conducted.

The environment where* H. hamelinensis* was isolated, Shark Bay, is prone to high salinity, exposed ultraviolet radiation and high temperatures leading to desiccation [36]. Therefore, it is likely that* H. hamelinensis* would need to possess various strategies to cope with such stresses, and recent studies have revealed several mechanisms this archaeon utilizes to cope with both high saline and UV stress [12, 13]. In terms of saline stress, most halophilic Archaea are known to import potassium ions as the primary osmoadaptive strategy [12, 37]. Interestingly, recent studies indicated that no potassium was taken up by* H. hamelinensis*, either after growth at both low and high salt concentrations [12]. Upon analysis of the genome of* H. hamelinensis* in the present study, however, it was found that a putative potassium uptake protein TrkH was present; however no homologs to KdpB were identified. Other putative potassium uptake proteins, such as TrkA and several others from the potassium efflux system, were identified (Table 2), although no complete pathway for potassium uptake was detected within the* H. hamelinensis* genome. It is hypothesized that these putative proteins may be inactive due to the lack of K^+^ uptake seen previously [12]; however it is also possible they may be expressed under different conditions. Other research based on multidrug susceptibility suggested that resistance to antibiotics in microorganisms is associated with several factors, including efflux pumps [38]. Specifically, one study found that the disruption of the putative potassium uptake regulator, TrkA, leads to rifampicin resistance [39], as well as conferring resistance to other antibiotics such as novobiocin [40, 41]. Interestingly,* H. hamelinensis* displays sensitivity to both rifampicin and novobiocin [10]; however it remains to be determined whether the putative TrkA present in this organism plays any role in its observed antibiotic sensitivities.

Early studies have also shown that halophilic enzymes exhibit highly polar surfaces in order to remain in solution [41], and comparative studies have shown that in particular ribosomal proteins from moderate halophiles often have a higher content of acidic amino acids than did the comparable proteins from* E. coli* and other nonhalophiles [42]. Therefore cytoplasmic proteins of halophiles often reveal high ratios of acidic amino acids, particularly glutamate and aspartate residues. A presence of high ratios of acidic amino acids was also observed in the genome of* H. hamelinensis* in the present study through codon prediction analyses, suggesting this feature may correlate with its ability to tolerate a high saline environment.

Glycine betaine accumulation has been shown to be the major adaptive response to osmotic stress in* H. hamelinensis* [12] and the presence of several glycine betaine uptake genes were confirmed in the present study. In addition, other solutes involved in osmoadaptive strategies in this archaeon include trehalose, glutamate, and proline [12]. Levels of trehalose were found to oscillate depending on the levels of glycine betaine present in the cell [12]; therefore the organism is likely able to synthesize and degrade this sugar depending on the level of other compatible solutes present in the cell and/or microenvironment. The high frequency of abundance displayed for codons that code for proline, glycine, and glutamic acid discovered in the present study in the* H. hamelinensis* in the genome further supports osmotolerance.* H. hamelinensis* can potentially use glycine betaine not only as an osmotic stabilizer but also as a carbon and energy source. An early study was carried out on a number of haloalkaliphilic isolates from Mono Lake, California, which grew on glycine betaine by sequential demethylation via dimethylglycine to sarcosine, later excreting glycine betaine into the medium [43]. At high salinities, glycine betaine catabolism was suppressed and the compound was taken up solely to serve as an osmotic solute, thereby allowing extension of the salt range, enabling growth in 2.5–3.5 M NaCl [12, 43]. Another study carried out on the Shark Bay stromatolites reported the synthesis of glycine betaine as a compatible solute in cyanobacteria [44], and it has been suggested that Archaea and cyanobacteria coexist in these systems through aspects of metabolic cooperation involving compatible solutes [12]. However further work is needed to clarify the exact role of glycine betaine in* H. hamelinensis* physiology.

Bacteriorhodopsin, halorhodopsin, and bacterioruberin are unique energy transducers involved in energy acquisition [25, 45]. These energy transducers are signature to most halophiles; however there were no homologs to these systems detected here in* H. hamelinensis*. This was intriguing, since cultures of this organism display a bright red-pink color [12], characteristic of these pigments. Furthermore earlier studies employing Raman spectroscopy confirmed the presence of bacterioruberin in* H. hamelinensis* [46], indicating further work is needed to deduce whether genes involved in the synthesis of these pigments are less conserved than expected or are present as genes encoding unknown or hypothetical proteins.

To further distinguish the* H. hamelinensis* from other organisms, analyses were conducted here to review the composition and representation of its COG categories.* H. hamelinensis* COG categories with the largest number of PCGs were surprisingly also the overrepresented COG categories within* H. hamelinensis*. The overrepresented COG categories consisted of amino acids transport and metabolism, carbohydrate transport and metabolism, and general function prediction only. These mostly consisted of PCGs relating to nutrient metabolism. Other overrepresented COG categories were energy production/conversion and inorganic transport and metabolism. These categories consisted of PCGs of various enzymes such as ATP-synthases, dehydrogenases, and multiple ABC-transporter genes. These COG categories suggest potential adaptation of* H. hamelinensis* towards high nutrient uptake and synthesis, which would be beneficial as this archaeon is prone to a low nutrient environment [47]. The secondary metabolites biosynthesis, transport, and catabolism and signal transduction mechanism COG categories were also overrepresented in* H. hamelinensis*, when compared to other archaeal genomes. These categories consisted of putative genes for synthesis of coenzyme A, antibiotics such as novobiocin and streptomycin, and presence of stress response proteins, respectively. These suggest putative defence mechanism that may be present in* H. hamelinensis*.

*H. hamelinensis* is associated witha highly diverse microbial community [7], and this organism displays potentially unique genome characteristics not present in many Archaea. To test the importance of horizontal gene transfer on* H. hamelinensis*, various detailed bioinformatics analyses were conducted. Interestingly there was significant similarity between* H. hamelinensis* and the bacterial lineage. There was 22.22% similarity between the* H. hamelinensis* genome and the phyla of* Actinobacteria*, which consists of many marine bacteria [48]. Additionally, there was 7.69% similarity between cyanobacteria and* H. hamelinensis*, which alludes to potential horizontal gene transfer between these organisms. Of further significance, four putative genes had >30% similarity between* H. hamelinensis* and dsDNA viruses genomes, elucidating a possible function of viral DNA being involved in gene transfer. Most of the bacterial phyla indicated <1% gene hits with the* H. hamelinensis* genes; therefore it is difficult to conclude based on this evidence that HGT was prevalent in the genome. Despite this, the apparent lack of CRISPR-associated genes in* H. hamelinensis* is consistent with a recent study indicating these predicted genes do not seem to be present in all haloarchaea [49]. Although it is possible other genes could be encoded for in some of the unknown/hypothetical genes of the* H. hamelinensis* genome, the apparent lack of these systems suggests this organism may be thus amenable to HGT, a trait which may be of critical importance in its specialized niche in the Shark Bay stromatolites.

## 5. Conclusions

**Figure 3:**
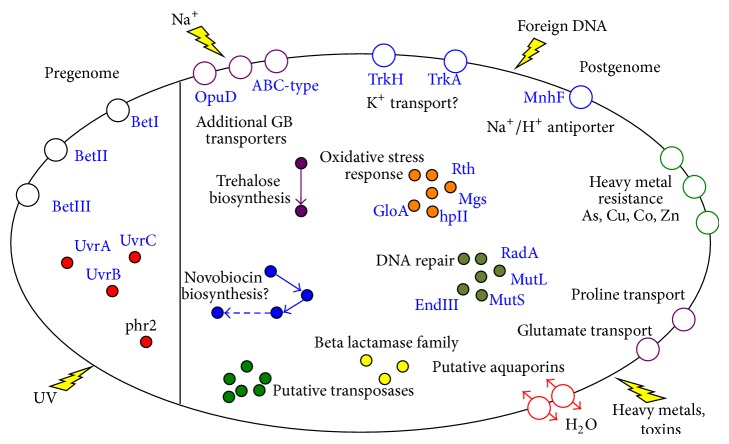
Schematic representation of some of the putative adaptive traits present in* H. hamelinensis*. The traits identified pregenome is indicated on the left, and those elucidated postgenome in the present study are indicated on the right.

This study reports the detailed genome analyses of* H. hamelinensis*, a novel archaeon, first isolated from modern stromatolites. Early studies carried out indicated the putative presence of three distinct and unique glycine-betaine transporters BetI, BetII, and BetIII (unpublished data) and the presence of a bacterial-like nucleotide excision repair (NER) system, containing UvrA, UvrB, and UvrC genes, as well as a photolyase [12, 13] in the* H. hamelinensis* genome. Postgenome analysis presented here revealed a range of other potential stress response mechanisms, and Figure 3 summarises some of the key adaptive responses identified through analyses in the current study. It also highlights the advantages of high-throughput sequencing and comparative genomics to build on pregenome sequencing work. In addition to DNA repair mechanisms and osmotic response, there were selective putative genes for heavy metal resistance such as arsenic. A recent study has suggested there may be a link between salinity and heavy metal uptake in some haloarchaea [50], and further work at the transcript and growth level may help elucidate the importance of these mechanisms in* H. hamelinensis*.

It is important to note that the genome analyses presented here describe the potential for a given function, and future detailed analyses at the gene and/or protein expression levels are required to definitively attribute the traits delineated in the this investigation. Such studies may ultimately help elucidate the role of this domain of life in these evolutionarily significant ecosystems.

## Figures and Tables

**Table 1:** General features of the *H. hamelinensis* genome.

General features	Number
Size (bp)	**3133046**
GC%^1^	**66.08**
RNA genes	**48**
rRNA genes	3
5 S rRNA	1
16 S rRNA	1
23 S rRNA	1
tRNA genes	45
DNA scaffolds	223
DNA coding bases	2529448
*Protein coding genes (PCG): *	**3150**
PCG with known and putative function (%)	68.67
PCG with enzymes (%)	22.39
PCG with COGs (%)	65.13
PCG with Pfam (%)	65.98
PCG with TIGRfam (%)	19.48
PCG with InterPro (%)	87.12
PCG coding signal peptides (%)	10.07
PCG coding transmembrane proteins (%)	22.48
PCG in paralog clusters (%)	20.04
Fused Protein coding genes	219
COG clusters	1177
KOG clusters	635
Pfam clusters	1207
TIGRfam clusters	539

^1^GC percentage shown as count of G's and C's divided by a total number of G's, C's, A's, and T's. COGs—Clusters of Orthologous Groups of proteins; Pfam—protein family database; TIGRFAMs—resource consisting of curated multiple sequence alignments, Hidden Markov Models for protein sequence classification, and associated information designed to support automated annotation of proteins; KOG—euKaryotic Orthologous Groups.

**Table 2:** Putative osmoadaptive genes identified in *H. hamelinensis* genome.

Gene name	Gene ID
Probable glycine betaine transporter protein (Bet III)	2502299791

Glycine betaine transporters OpuD	2502299823

Glycine betaine transporter	2502299930

ABC-type proline/glycine betaine permease component	2502300305

Glycine betaine ABC transporter, ATP-binding protein	2502300306

ABC-type proline/glycine betaine permease component	2502300307

Probable glycine betaine transport protein	2502301633

Potassium uptake protein TrkH	fig∣332168.3.peg.1799

TrkA system potassium uptake protein	ffig∣332168.3.peg.105

Potassiumchannel protein	2502299500

Kef-type K^+^ transport systems	fig∣332168.3.peg.334

**Table 3:** Putative genes coding for heavy metal resistance in *H. hamelinensis*.

Gene name	Gene ID
Arsenical pump-driving ATPase	fig∣332168.3.peg.1469

Arsenate reductase (EC 1.20.4.1)	fig∣332168.3.peg.2242

Arsenate reductase (EC 1.20.4.1)	fig∣332168.3.peg.3119

Arsenical-resistance protein ACR3	fig∣332168.3.peg.3118

DNA gyrase subunit A (EC 5.99.1.3)	fig∣332168.3.peg.722

DNA gyrase subunit B (EC 5.99.1.3)	fig∣332168.3.peg.721

Aminoglycoside N6′-acetyltransferase (EC 2.3.1.82)	fig∣332168.3.peg.687

Copper-translocating P-type ATPase (EC 3.6.3.4)	fig∣332168.3.peg.2377

Copper-translocating P-type ATPase (EC 3.6.3.4)	fig∣332168.3.peg.2190

Cobalt-zinc-cadmium resistance protein CzcD	fig∣332168.3.peg.2193

Cobalt-zinc-cadmium resistance protein	fig∣332168.3.peg.471

Permease of the drug/metabolite transporter (DMT) superfamily	fig∣332168.3.peg.1282

Permease of the drug/metabolite transporter (DMT) superfamily	fig∣332168.3.peg.2762

Permease of the drug/metabolite transporter (DMT) superfamily	fig∣332168.3.peg.2832

Permease of the drug/metabolite transporter (DMT) superfamily	fig∣332168.3.peg.2837
